# SARS-CoV-2 Seroprevalence in Healthcare Workers in Germany: A Follow-Up Study

**DOI:** 10.3390/ijerph18094540

**Published:** 2021-04-25

**Authors:** Johannes Korth, Benjamin Wilde, Sebastian Dolff, Jasmin Frisch, Michael Jahn, Adalbert Krawczyk, Mirko Trilling, Leonie Schipper, Sebastian Cordes, Birgit Ross, Monika Lindemann, Andreas Kribben, Ulf Dittmer, Oliver Witzke, Anke Herrmann, Olympia Evdoxia Anastasiou

**Affiliations:** 1 Department of Nephrology, University Hospital Essen, University of Duisburg-Essen, Hufelandstr. 55, 45147 Essen, Germany; Benjamin.wilde@uk-essen.de (B.W.); michael.jahn@uk-essen.de (M.J.); andreas.kribben@uk-essen.de (A.K.); 2 Department of Infectious Diseases, West German Centre of Infectious Diseases, University Hospital Essen, University of Duisburg-Essen, Hufelandstr. 55, 45147 Essen, Germany; sebastian.dolff@uk-essen.de (S.D.); jasmin.frisch@stud.uni-due.de (J.F.); adalbert.krawczyk@uk-essen.de (A.K.); leonie.schipper@uk-essen.de (L.S.); oliver.witzke@uk-essen.de (O.W.); 3 Institute for Virology, University Hospital Essen, University of Duisburg-Essen, Virchowstr. 179, 45147 Essen, Germany; mirko.trilling@uk-essen.de (M.T.); ulf.dittmer@uk-essen.de (U.D.); anke.herrmann@uk-essen.de (A.H.); olympiaevdoxia.anastasiou@uk-essen.de (O.E.A.); 4 Department of Pneumology, University Hospital Essen, University of Duisburg-Essen, Tüschener Weg 40, 45239 Essen, Germany; sebastian.cordes@rlk.uk-essen.de; 5 Hospital Hygiene, University Hospital Essen, University Duisburg-Essen, Hufelandstr. 55, 45147 Essen, Germany; birgit.ross@uk-essen.de; 6 Institute for Transfusion Medicine, University Hospital Essen, University of Duisburg-Essen, 45147 Essen, Germany; monika.lindemann@uk-essen.de

**Keywords:** SARS-CoV-2, healthcare workers, COVID-19, seroprevalence, hygiene standards

## Abstract

SARS-CoV-2 is a worldwide challenge for the medical sector. Healthcare workers (HCW) are a cohort vulnerable to SARS-CoV-2 infection due to frequent and close contact with COVID-19 patients. However, they are also well trained and equipped with protective gear. The SARS-CoV-2 IgG antibody status was assessed at three different time points in 450 HCW of the University Hospital Essen in Germany. HCW were stratified according to contact frequencies with COVID-19 patients in (I) a high-risk group with daily contacts with known COVID-19 patients (n = 338), (II) an intermediate-risk group with daily contacts with non-COVID-19 patients (n = 78), and (III) a low-risk group without patient contacts (n = 34). The overall seroprevalence increased from 2.2% in March–May to 4.0% in June–July to 5.1% in October–December. The SARS-CoV-2 IgG detection rate was not significantly different between the high-risk group (1.8%; 3.8%; 5.5%), the intermediate-risk group (5.1%; 6.3%; 6.1%), and the low-risk group (0%, 0%, 0%). The overall SARS-CoV-2 seroprevalence remained low in HCW in western Germany one year after the outbreak of COVID-19 in Germany, and hygiene standards seemed to be effective in preventing patient-to-staff virus transmission.

## 1. Introduction

The severe acute respiratory syndrome coronavirus-2 (SARS-CoV-2) is a worldwide challenge for healthcare systems. The number of confirmed cases worldwide increased to 127 million on 29 March 2021, with the Robert Koch Institute reporting 2.7 million cases and over 75,000 deaths in Germany. The University Hospital Essen was designated the primary admission hospital for COVID-19 patients for an area of around five million residents. Healthcare workers (HCW) are vulnerable to infection due to frequent and close contact with COVID-19 patients [1,2], as the primary route of transmission is from person to person [3]. After infection, a seroconversion with detection of IgG antibodies is observed after 11 to 14 days [4]. This study reports a longitudinal observation of the seroprevalence course of SARS-CoV-2 at three periods (March–May, June–July, and October–December) in 2020 in 450 healthcare workers of the university Hospital Essen in Germany. In addition, this study evaluates if HCW with daily COVID-19 patient contact are at greater risk for SARS-COV-2 infection overtime compared with HCW with less frequent contact with COVID-19 patients.

## 2. Materials and Methods

HCW of the University Hospital Essen were asked to take part in the study. Participants were grouped according to the frequency of contact with COVID-19 patients: (I) a high-risk group with daily contact with COVID-19 patients on designated wards and on intensive care units, (II) an intermediate-risk group with daily contact with patients without known COVID-19, and (III) a low-risk group without daily patient contact. The three periods of sampling were (a) 25 March 2020 until 27 May (b) 1 June until 24 July and (c) 1 October until 15 December 2020. SARS-CoV-2 seroprevalence was evaluated using an approved and CE-labelled anti-SARS-CoV-2 IgG ELISA (Euroimmun Medizinische Labordiagnostika, Lübeck, Germany), which recognizes IgG antibodies specifically binding to the SARS-CoV-2-encoded spike protein subunit 1 (S1). To compare the estimated seroprevalence in HCW with the incidence of the area’s population, the SARS-CoV-2 incidence of the district of Essen was prompted from the Robert-Roch-Institut (https://survstat.rki.de (accessed on 22 February 2021)). In addition, a plaque reduction neutralization test was performed on 18 of 19 participants being tested for SARS-CoV-2 IgG in the ELISA to evaluate the serum neutralizing activity as described before [5]. The course of the signal-to-cut-off ratio (S/CO) of participants who tested positive in the ELISA and the course of the neutralizing test were further analyzed at baseline and during the observational period.

According to the manufacturer’s recommendations for the ELISA, an S/CO ratio of <0.8 was considered negative, ≥0.8 to <1.1 borderline, and ≥1.1 positive. The ELISA has a sensitivity of 94.4% for samples taken 10 days after the first symptoms or SARS-CoV-2 diagnosis without taking borderline results into account, according to the manufacturer (Euroimmun, anti-SARS-CoV.2 ELISA IgG, test instructions), and a specificity of 99.6%. Thus, false positive results are expected to be very rare, while false negative results may occasionally occur, albeit infrequently. Strategies to avoid patient-to-staff transmission included testing the patients with qRT-PCR before hospital admission and not allowing patients to have visitors. For healthcare professionals to deliver safe care [6] on designated COVID-19 wards (high-risk group), a local hygiene standard was prepared including personal protective equipment (PPE) consisting of respiratory protection, N95 Respirators, a disposable coat, waterproof gowns, single-use gloves, and a face shield. Ventilated COVID-19 patients had to be equipped with a heat and moisture exchanger/bacterial filter. Staff on wards without known or suspected COVID-19 patients (intermediate-risk group) adhered to basic hygiene standards according to the WHO. In addition, all staff members had to wear surgical masks starting on 14 April 2020. All hospital members had to adhere to hand hygiene measures according to the World Health Organization Guidelines. Complying with the hygiene standards was essential according to the professional liability laws. In an article by Heinz, patients have in general the burden of proof in cases of an alleged hygiene requirement violation [7] (§ 23 Abs. 3 Satz 2 IfSG). In fact, patient- interest groups demand a reversal of the burden of proof, with the hospital having to prove that the hygiene standards were upheld. As in other European countries, a penal shield does not currently exist [8]. Fisher’s exact test was used to compare results between groups. Informed consent was obtained from all participants. Partial baseline results for 316 HCW with sampling between 25 March 2020 and 21 April 2020 have been previously published [9].

## 3. Results

Overall, 450 HCW participated in the study and took part in the first survey (296 nurses, 115 physicians, and 39 other employees, such as secretaries and lab assistants). Subjects’ characteristics at baseline are listed in Table 1.

### 3.1. Seroprevalance

#### 3.1.1. First Observational Period: March–May 2020

**Figure 1:**
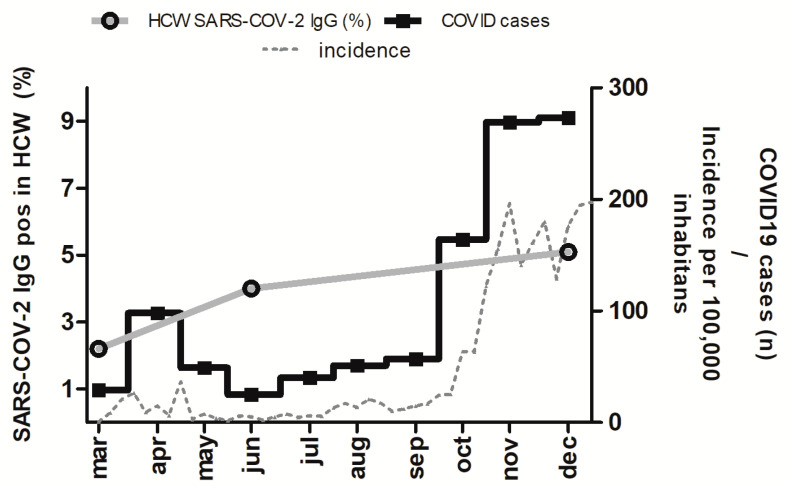
Frequency of healthcare workers testing positive for SARS-COV-2 IgG (grey); the COVID19 cases (black) treated at the University Hospital Essen; the COVID-19 incidence per 100,000 inhabitants in the Essen district from March 2020 (week 10) to December 2020 (week 53). The data of the COVID-19 incidence in the district of Essen are publicly available from the Survstat@RKI 2.0 server. Robert Koch-Institut: SurvStat@RKI 2.0, https://survstat.rki.de, accessed on: 22 February 2021.

SARS-CoV-2 IgG antibodies were detected in 10 of 450 (2.2%) subjects (Figure 1). Of them, 6/338 (1.8%) belonged to the high-risk group and 4/78 (5.1%) to the intermediate-risk group. The seroprevalence was not significantly different between the high-risk group and the intermediate-risk group (odds ratio 0.33; 95%CI 0.09–1.21; *p* = 0.09). None of the low-risk group (n = 34) tested positive for SARS-CoV-2 IgG antibodies.

#### 3.1.2. Second Observational Period: June–July 2020

Follow-up samples were available for 349 of the 450 participants (77%). Four participants (all in the high-risk group) seroconverted. SARS-COV-2 IgG antibodies were detected in 14 of 349 (4%) (Figure 1); 10/260 (3.8 %) belonged to the high-risk group; 4/63 (6.3%) to the intermediate-risk group. The seroprevalence was not significantly different between the high-risk group and the intermediate-risk group (odds ratio 0.59; 95%CI 0.17–1.94; *p* = 0.38). None of the low-risk group (n = 26) tested positive for SARS-CoV-2 IgG antibodies.

#### 3.1.3. Third Observational Period: October–December 2020

Follow-up samples were available for 315 of the 450 participants (70%). Five participants (all high-risk group) seroconverted. SARS-CoV-2 IgG antibodies were detected in 16/315 (5.1%) (Figure 1). Of these, 13/236 (5.5%) subjects belonged to the high-risk group, and 3/49 (6.1%) belonged to the intermediate-risk group. The seroprevalence was not significantly different between the high-risk group and the intermediate-risk group (odds ratio 0.89; 95%CI 0.24–3.26; *p* = 0.86). None of the low-risk group (n = 30) tested positive for SARS-CoV-2 IgG antibodies. Two participants (1× high risk group, 1× intermediate risk group) who tested positive in the first and second observational period tested negative for SARS-CoV-2 IgG in the third observational period. One staff member (high-risk group) who tested positive in the first and second observational period did not participate in the last follow up. There was no difference in the number of infections between physicians and nurses (odds ratio 1.13; 95%CI 0.42–3.12; p = 0.80).

**Figure 2:**
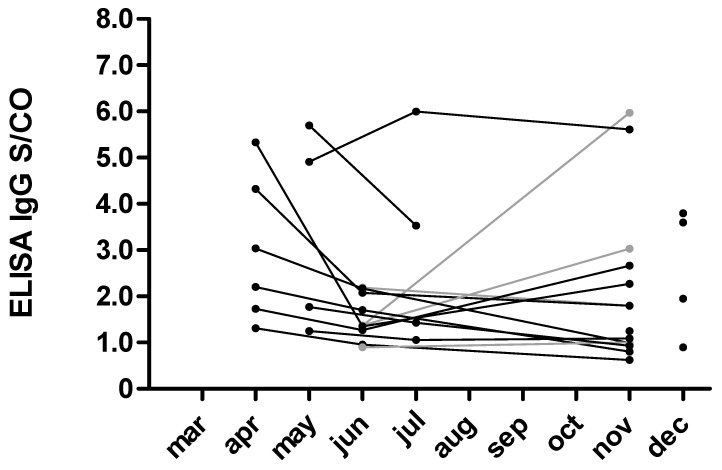
IgG ELISA signal to cutoff (S/CO) in healthcare workers testing positive for SARS-CoV-2 IgG. The black line indicates the follow-up of participants who tested positive for SARS-CoV-2 IgG during the first observational period. The grey lines indicate the follow-up of participants who tested positive during the second observational period. The triangles illustrate the S/CO’s of participants who tested positive within the third observational period.

SARS-CoV-2 IgG follow-up data from the 10 participants who tested positive in the first observational period were available with a mean follow up of 4.0 ± 1.24 months (Figure 2). The S/CO for the SARS-CoV-2 IgG antibodies decreased by −0.41 ± 2.06. The two previously mentioned seroreverting participants tested negative for SARS-CoV- IgG by ELISA at the last visit after 4 and 5 months, respectively. However, neutralizing antibodies could still be detected using the neutralization assay.

### 3.2. Neutralization Antibodies

We tested the serum neutralizing activity in 18 of the 19 participants who tested positive for SARS-CoV-2 IgG antibodies. In 3 of the 18 (17%) participants, no neutralizing antibodies were detectable.

**Figure 3:**
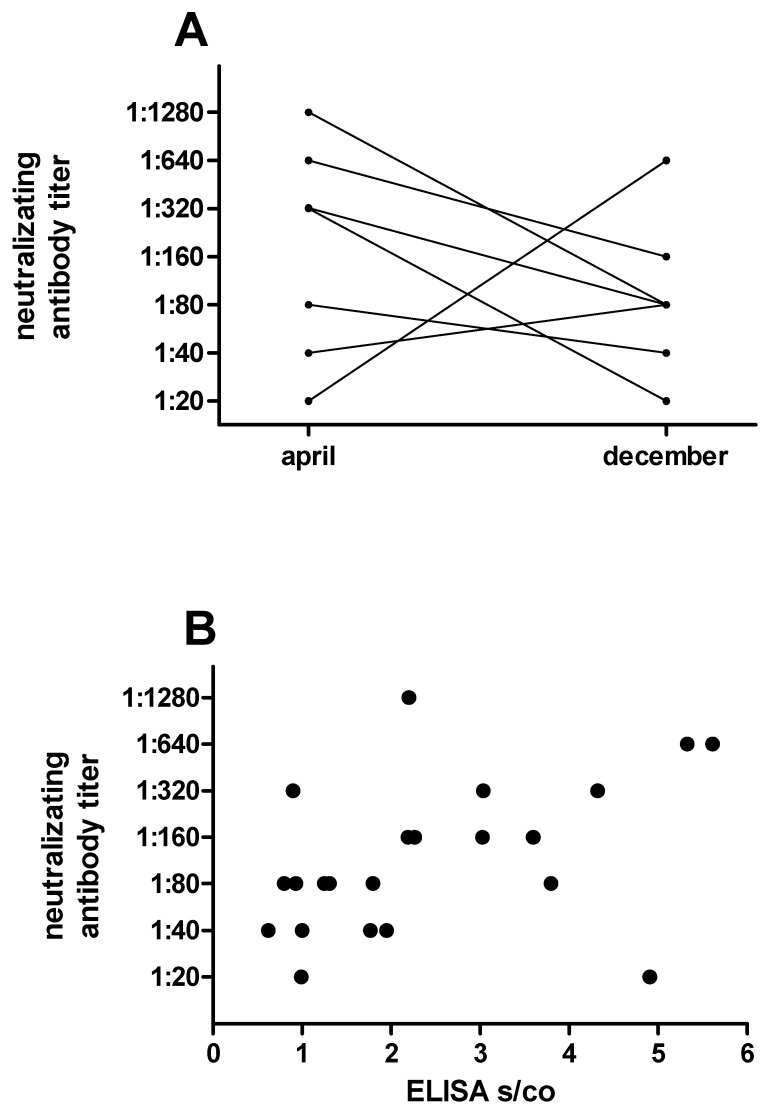
Distribution and follow up of neutralizing antibody titers in healthcare workers. (**A**) Follow up of 5 patients with detectable neutralizing antibody titers in April and December 2020. The undetectable SARS-CoV-2 neutralizing antibody titer of two participants are not illustrated. (**B**) Correlation of EUROIMMUN SARS-CoV-2 IgG ELISA signal to cutoff (S/CO) ratio and neutralization antibody titers.

Follow up data was available for nine patients after 4.5 ± 0.72 months. In five participants, the specific IgG-titer decreased, in two others the titer increased, and two had no detectable neutralization antibody titers in either sample (Figure 3A). No follow up data was available for the third participant without neutralization activity. The neutralizing antibody titers and ELISA S/CO values (26 pairs) did not correlate well (r = −0.26, *p* = 0.20, Figure 3B).

### 3.3. Symptoms

SARS-CoV-2 IgG antibodies could be detected in 19 HCW during the observational period. Of these HCW, 17 of the 19 (89%, Table 2) provided information about recent symptoms in the past three months, including headache (9/19 47%), general malaise (8/19 42%), rhinitis (7/19 37%), anosmia (7/19 37%), cough (6/19 32%), sore throat (5/19 26%), fever (5/19 26%) and dyspnea (3/19 16%). Additionally, 4 of the 19 subjects (21%) had no typical symptoms at all. None of the subjects were hospitalized. The infection route could be reconstructed in 6 of the 19 HCW and revealed that three of the subjects were infected during vacation and three had suspected infection after unprotected COVID-19 exposure in March 2020. In 13 cases, the route of infection was unknown. In 11 of the 19 participants, PCR´s were performed between March and December 2020. Of these, 7 of the 11 (63%) participants had positive PCR results.

## 4. Discussion

The aim of this longitudinal observational study was to determine the SARS-CoV-2 seroprevalence in HCW during the ongoing COVID-19 pandemic and to observe if HCW with direct COVID-19 contact are at greater risk for SARS-CoV-2 infection compared to other hospital staff members. SARS-CoV-2 IgG were measured during three different periods (a. March–May, b. June–July and c. October–December). A total of 450 HCW participated in the study and 1114 samples were tested for SARS-CoV-2 IgG antibodies. During 2020, more than 1000 COVID-19 patients were treated in the designated COVID-19 units. During the first observational period, which corresponded to the duration of the first pandemic wave, the overall seroprevalence was 2.2%. During the second observational period, the seroprevalence went up sharply to 4.0%, although the COVID-19 incidence in the general population of the district of Essen was at an all-time low after the first COVID-19 wave. During the third observational period, the seroprevalence amounted to 5.1% of the tested HCW, increasing only slightly compared to the second observational period. In contrast, the COVID-19 incidence in the community and the number of COVID-19 treated patients increased sharply (Figure 1). The sharp increase of the seroprevalence in HCW after the first wave may be based on a delayed diagnosis of past infections during the first wave. The following stable HCW seroprevalence despite the rapidly increasing incidence of the population may be a consequence of the obligatory surgical mask for staff members starting 14 April 2020. In addition, knowledge about COVID-19 tends to be high in health care workers, which may indirectly have a positive effect on limiting transmission [10,11]. The SARS-CoV-2 seroprevalence in blood donors in North Rhine–Westphalia from March to June 2020 was 0.9% [12], while a community-based prospective cohort study in people aged 30 years and above in a North Rhine–Westphalia city, Bonn, performed between 24 April and 30 June 2020, showed a seroprevalence of 1% [13], which is somewhat lower but comparable to the seroprevalence we observed in our cohort between March and May 2020. Unfortunately, data regarding the SARS-CoV-2 seroprevalence of the district´s population are not available at the time being. Consistent with our previous results, [9] there was a numerical difference in the seroprevalence of the high-risk group (1.8%; 3.8%; 5.5%) vs. the intermediate-risk group (5.1%; 6.3%; 6.1%) during the first observational phase; the difference, however, did not reach statistical significance. In the second (and even more in the third) observational period, the seroprevalence of the two groups converged, which can be attributed to the fact that infections after the first observational period occurred mainly in the high-risk group. There was no difference between physicians and nurses in the number of infections. An interesting study from Nioi et al., in which the deaths and specialization of each deceased physician in Italy was analyzed, observed that the most affected doctors were general practitioners and dental practitioners [14]. The authors supposed a shortage of protective supplies to be the reason for the elevated infection rates and deaths. Even with the observed increase of the COVID-19 seroprevalence in the high-risk group at the University Hospital Essen, the seroprevalence was low compared to several other centers in Europe and the world, as shown in a metanalysis by Chen et al. [15]. We should stress, however, that the metanalysis included just two studies with low-risk-HCW from Europe, and the overall seroprevalence of 3.1% was based on data from just one study performed in March and April of 2020 in Western Germany [16]. More strikingly, a study from Southern Germany evaluating the COVID-19 seroprevalence in HCW showed a higher seroprevalence in HCW with patient contact (n = 2387) vs. a low-risk group (n = 1,850) without patient contact (15.1% vs. 3.7%) [17]. In addition, members from the non-COVID-19 ward had lower SARS-CoV-2 IgG detection rates in comparison to COVID-19 wards (14.4% vs. 26.2%), which was much higher compared to our results. In another study with 2,149 HCW from Sweden performed from April to May, 19.1% tested positive [18]. Other studies, however, showed considerably lower seroprevalence rates amongst HCW. A study from Northern Germany performed in July 2020 observed an overall seroprevalence of 1.8% in 1,253 HCW. They could not detect a significant difference in HCW directly involved in patient care compared to other hospital employees [19]. A longitudinal study of 679 HCW conducted in Milan, Italy observed similar seroprevalence rates as the current study: March 0.5%; April 5.4%; May 5.9% [20]. Finally, a metanalysis published in October 2020 concluded that the COVID-19 seroprevalence in HCW in Europe is around 8.5% [21]. The authors summarized that the seroprevalence among HCW varies greatly and that adherence to infection prevention and control measures, personal protective equipment, early recognition, identification, and isolation of HCW infected with SARS-CoV-2 are imperative to decreasing the risk of SARS-CoV-2 infection. Indeed, our results underline the importance of infection prevention and control measures. The low seroprevalence at baseline and the slight increase in COVID-19 seroprevalence overtime in our HCW, despite a considerable increase in the number of treated COVID-19 patients and in the COVID-19 incidence in the community, indicate that the hygiene measures taken to limit COVID-19 transmission in the wards were effective. One of the most effective strategies seems to be the obligatory facemasks, since N95 respirators prevent the passage of 99.8% of particles larger than 0.1 μm in practice [22], and the SARS-CoV-2 virus transmissions occurs through respiratory droplets or aerosols of 1–5 μm or larger [23]. Although, direct comparison of surgical masks and N95 respirators revealed no difference in other viral respiratory infections in a metanalysis of Bartoszko and colleagues [24], the U.S Centers for Disease Control and Prevention (CDC) and the European Centre for Disease and Prevention (ECDC) recommend the use of N95 respirators for non-aerosol-generating procedures over the surgical masks. The longitudinal course of SARS-CoV-2 antibodies in HCW after infection was similar to that reported for other patients [25]. An interesting observation was that neutralization antibodies could still be detected in two participants with undetectable IgG antibodies in ELISA after seroreversion. Considering the increased risk for SARS-CoV-2 infection in HCW and its potentially disastrous repercussions for the treated patients of HCW, the prioritized vaccination of HCW in Germany is reasonable (www.rki.de/covid-19-impfen (accessed on 22 February 2021)). Considering the low COVID-19 seroprevalence, testing for COVID-19 antibodies before vaccination does not seem efficient. Further studies have to investigate the duration of immunity after vaccination of the current candidates [26]. In addition, evaluation and monitoring of the immunity in serological non-responder HCW after vaccination will be interesting since the majority of patients after SARS-CoV-2 infection without detectable antibodies show a specific T lymphocyte-based immunity [27]. An important limitation of the study is its convenient sampling design; any HCW in a given sector could volunteer to participate in the study. It is conceivable that HCW with a COVID-19 diagnosis or previous symptoms consistent with COVID-19 would be more interested in a measurement of their serum COVID-19 antibodies. Another limitation is the lack of infections in the low-risk group. This could be attributed to an intense awareness of the low-risk group participants, since they could witness the risk of infection while working at a designated COVID-19 hospital even without patient contact. However, our study also has strengths. It presents data on the longitudinal course of COVID-19 seroprevalence based on a largely consistent cohort of HCW. In addition, the data are drawn from a “prevaccination” period, from the beginning of the pandemic to the time before the first vaccinations, and may thus provide a useful contrast to future data acquired post-vaccination.

## 5. Conclusions

In conclusion, the overall seroprevalence of healthcare workers of the University Hospital Essen was 5.1%. Healthcare workers on COVID-19 wards were not at a greater risk for SARS-CoV-2 infection in comparison with non-COVID-19 wards. The local hygiene standards seemed to be effective over time in limiting patient-to-staff transmission at the COVID-19 wards.

## Figures and Tables

**Table 1:** Participant’s characteristics, subject characteristics, SD standard deviation, SARS-CoV-2 severe acute respiratory syndrome coronavirus 2, IgG immunoglobulin G, S/co signal-to-cut-off ratio. HCW healthcare workers.

	High-Risk		Intermediate-Risk		Low-Risk		Overall	
	n	%	n	%	n	%	n	%
**HCW**	338	75%	78	17%	34	8%	450	100%
**female**	220	65%	49	63%	25	74%	294	65%
**age (±SD)**	36.6 ± 10.7		42.6 ± 11.4		42.2 ± 13.0		38.1 ± 11.3	
**profession**								
**nurse**	246	73%	50	64%	0	0%	296	66%
**physician**	84	25%	22	28%	9	26%	115	26%
**lab assistant**	0	0%	0	0%	20	59%	20	4%
**other**	8	2%	6	8%	5	15%	19	4%

**Table 2:** Symptoms of healthcare workers who tested positive for SARS-CoV-2 IgG antibodies; n number.

Symptoms	Healthcare Workes					
	Positive (n, %)		Negative (n, %)		Unknown (n, %)	
**headache**	9	(47%)	8	(42%)	2	(11%)
**general malaise**	8	(42%)	9	(47%)	2	(11%)
**anosmia**	7	(37%)	10	(53%)	2	(11%)
**rhinitis**	7	(37%)	10	(53%)	2	(11%)
**cough**	6	(32%)	11	(58%)	2	(11%)
**fever**	5	(26%)	12	(63%)	2	(11%)
**sore throat**	5	(26%)	12	(63%)	2	(11%)
**dyspnoea**	3	(16%)	14	(74%)	2	(11%)
**asymptomatic**	4	(21%)	13	(68%)	2	(11%)
**hospitalization**	0	(0%)	17	(89%)	2	(11%)

## Data Availability

The data that support the findings of this study are available from the 164 corresponding authors upon reasonable request.
